# Development of cytoplasmic male sterile lines and restorer lines of various elite Indica Group rice cultivars using CW-CMS/*Rf17* system

**DOI:** 10.1186/s12284-019-0332-8

**Published:** 2019-09-18

**Authors:** Kinya Toriyama, Tomohiko Kazama, Tadashi Sato, Yoshimichi Fukuta, Masaaki Oka

**Affiliations:** 1 Graduate School of Agricultural Science, Tohoku University, 468-1 Aramaki Aza Aoba, Aoba-ku, Sendai, Miyagi 980-8572 Japan; 2 Tropical Agriculture Research Front, Japan International Research Center for Agricultural Sciences, Ishigaki, Okinawa, 907-0002 Japan; 3 Miyagi University of Education, 149, Aramaki-aza-Aoba, Aobaku, Sendai, Miyagi 980-0845 Japan

**Keywords:** Cytoplasmic male sterility, Restorer of fertility, Hybrid rice

## Abstract

**Background:**

A cytoplasm of CW-type cytoplasmic male sterile (CMS) line is derived from *Oryza rufipogon* strain W1 and fertility is restored by a single nuclear gene, *Rf17.* We have previously reported that CW-CMS were effective for breeding CMS lines of Indica Group rice cultivars, IR 24 and IR 64. The applicability of this CW-CMS/*Rf17* system to produce other elite Indica Group rice cultivars with CMS was explored.

**Findings:**

Out of seven elite Indica Group rice cultivars, complete CMS lines were obtained for six cultivars: NSIC Rc 160, NSIC Rc 240, Ciherang, BRRI dhan 29, NERICA-L-19, and Pusa Basmati. The fertility of these six lines was restored when *Rf17* was present. A CMS line was not obtained for the cultivar Samba Mahsuri.

**Conclusions:**

The CW-CMS/*Rf17* system will be useful to produce CMS lines and restorer lines of various elite Indica Group rice cultivars.

## Findings

Hybrid rice has an average yield advantage of 15% to 20% over inbred cultivars. Most commercial hybrid rice has been developed based on a three-line system, namely A (CMS line), B (maintainer line), and R (restorer line). B lines must lack any *Rf* genes and the seeds of CMS lines are multiplicated by crossing A x B. F_1_ hybrid seeds are produced by crossing A x R. The resulting F_1_ hybrid plants are fertile, because an *Rf* gene is provided by the R line. The most predominantly utilized CMS system is known to employ wild abortive-type CMS (WA-CMS), which has accounted for about 90% of the three-line hybrids produced in China and 100% of the hybrids developed outside of China (Sattari et al. 2007; Huang et al. 2014). A major technical handicap in the development of hybrid rice using WA-CMS is a limited source of maintainer lines, as many Indica Group elite cultivars are known to carry restorer genes for WA-CMS lines; thus, they cannot be used as maintainer lines (Virmani 1994). For example, IR 24 and IR 64 are restorer lines for WA-CMS lines, and they are used as male parents for hybrid seed production (Jing et al. 2001; Cai et al. 2013).

We obtained CMS lines of IR 24 and IR 64 when we employed the Chinese wild rice (CW)-type CMS/*Restorer of fertility 17* (*Rf17*) system (Toriyama and Kazama 2016). CW-CMS is derived from *Oryza rufipogon* Griff. strain W1 (Katsuo and Mizushima 1958). A CMS-associated mitochondrial gene is *orf307* (Fujii et al. 2010). Pollen grains of the CW-CMS lines IR 24, IR 64, and a Japonica Group cultivar, Taichung 65, accumulate starch and look morphologically normal but lack germination ability (Fujii and Toriyama 2005; Toriyama and Kazama 2016). Fertility of the CW-CMS lines is gametophytically restored by a single nuclear gene, *Rf17*, which is identified to be a reduced expression allele of *RETROGRADE-REGULATED MALE STERILITY* (Fujii and Toriyama 2009; DDBJ accession number AB481199). To broaden the combination of male and female parents for hybrid rice production, seven elite Indica Group rice cultivars were tested for their acquisition of CMS by using the CW-CMS/*Rf17* system.

**Fig. 1:**
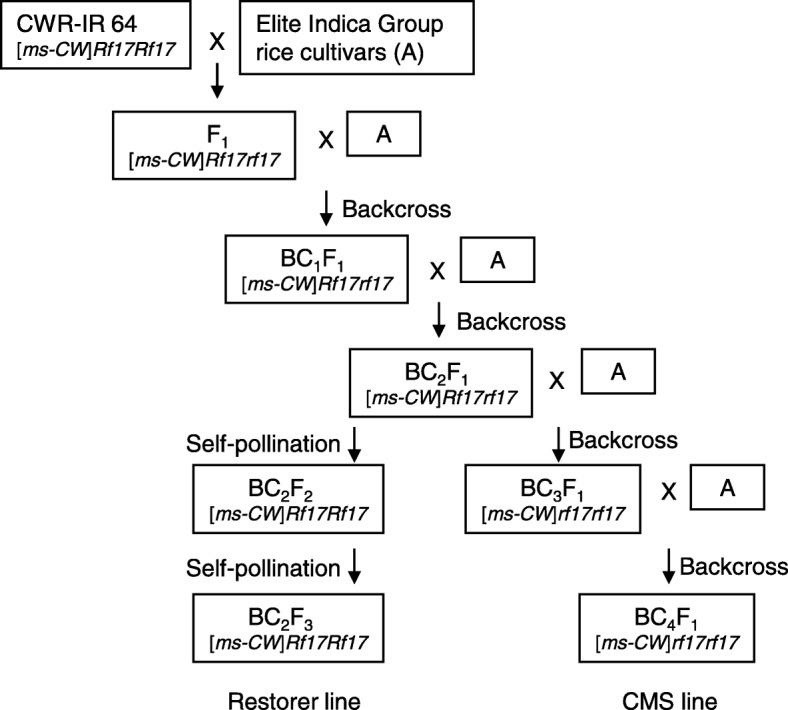
Flowchart of developing new CMS lines and restorer lines. [*ms-CW*] indicates the cytoplasm

The IR 64 nuclear background restorer line CWR-IR 64, which carried CW-cytoplasm and *Rf17Rf17* (Toriyama and Kazama 2016), were successively backcrossed with the elite Indica Group cultivars of NSIC Rc 160 (a high-quality eating cultivar), NSIC Rc 240 (a high-yielding cultivar) in the Philippines, Ciherang (a high-yielding cultivar) in Indonesia, BRRI dhan 29 (a high-yielding cultivar in Bangladesh), Pusa Basmati (an aromatic cultivar with short culm) in India, Samba Mahsuri (a high-yielding fine-grain rice cultivar) in India, and NERICA-L-19 (a high-yielding cultivar with the IR 64 genetic background) in Africa. A local Basmati line was also used as a recurrent parent, which was provided by the National Institute of Agrobiological Sciences Genebank (Tsukuba, Japan) as WRC42 (Kojima et al. 2005). The presence or absence of the *Rf17* allele was detected by a SNP in the promoter region 2286 bp upstream of the initiation codon; *Rf17* carried T, while *rf17* carried A (Fujii and Toriyama, 2009; Toriyama and Kazama 2016). Plants with *rf17rf17* were selected as CMS candidates, while plants with *Rf17Rf17* were selected as restorer candidates after the self-pollination of plants with *Rf17rf17* (Fig. 1).

The plants were grown in a biotron as previously described (Ohnishi et al. 2013). The filled and unfilled grains of bagged panicles were counted to calculate the seed setting rate. As shown in Table 1, the backcrossed lines with *rf17rf17* at generations BC_3_F_1_ and BC_4_F_1_ were completely sterile for NSIC Rc 160, NSIC Rc 240, Ciherang, BRRI dhan 29, and NERICA-L-19. Those at generations BC_2_F_1_ and BC_3_F_1_ were also completely sterile for Pusa Basmati. In contrast, the BC_3_F_1_ plants of Samba Mahsuri with *rf17rf17* set seeds segregating a plant with a higher seed setting rate of 79.6%. A progeny of this plant (BC_3_F_2_) also showed a higher seed setting rate of 77.8%.

**Table 1:** Seed setting rates (%) of backcrossed lines and pollen parents in 2017 and 2018

Initial female parent	Recurrent pollen parent	Backcrossed line								Pollen parent	
		*rf17rf17*				*Rf17Rf17*					
		2017 ^a)^		2018 ^b)^		2017 ^a)^		2018 ^b)^		2017 ^a)^	2018 ^c)^
CWR-IR 64	NSIC Rc 160	BC_3_F_1_	0	BC_4_F_1_	0	BC_2_F_2_	79.9	BC_2_F_3_	83.1	92.7	95.8
CWR-IR 64	NSIC Rc 240	BC_3_F_1_	0	BC_4_F_1_	0	BC_2_F_2_	88.7	BC_2_F_3_	75.2	92.8	81.3
CWR-IR 64	Ciherang	BC_3_F_1_	0	BC_4_F_1_	0	BC_2_F_2_	96.0	BC_2_F_3_	82.4	81.7	96.0
CWR-IR 64	BRRI dhan 29	BC_3_F_1_	0	BC_4_F_1_	0	BC_2_F_2_	75.7	BC_2_F_3_	73.1	87.1	80.9
CWR-IR 64	NERICA-L-19	BC_3_F_1_	0	BC_4_F_1_	0	BC_2_F_2_	65.1	BC_2_F_3_	56.3	77.1	71.1
CWR-IR 64 × Local Basmati BC_3_F_3_	Pusa Basmati	BC_2_F_1_	0	BC_3_F_1_	0	BC_1_F_2_	84.4	BC_1_F_3_	76.1	63.8	77.8
CWR-IR 64	Samba Mahsuri	BC_3_F_1_	79.6	BC_3_F_2_	77.8	BC_2_F_2_	74.7	BC_2_F_3_	85.9	79.6	ND
			44.2	ND	ND						
			40.7	ND	ND						

^a^Average of three bagged panicles of a single plant

^b^Average of three plants, each containing three bagged panicles

^c^Average of two plants, each containing three bagged panicles

The backcrossed lines of NSIC Rc 160, NSIC Rc 240, Ciherang, BRRI dhan 29, and NERICA-L-19 with *Rf17Rf17* at generations BC_2_F_2_ and BC_2_F_3_ showed higher seed setting rates comparable to those of each pollen parent, indicating that the fertility was recovered by the presence of *Rf17* (Table 1). The plants that were backcrossed with local Basmati three times (BC_3_F_3_) followed by the backcrossing with Pusa Basmati (BC_1_F_2_) also showed higher seed setting rates of approximately 80% in the presence of *Rf17* (Table 1).

Plants were also cultivated in an isolated glasshouse and a paddy field at Tropical Agriculture Research Front, Japan International Research Center for Agricultural Sciences located subtropical Ishigaki island from March to July, 2019, together with their pollen parents. Seed setting rates were evaluating using open pollinated panicles without bagging. For CMS lines, BC_4_F_1_ plants with *rf17rf17* of NSIC Rc 160, NSIC Rc 240, BRRI dhan 29, and NERICA-L-19 and Pusa Basmati are completely male sterile except for one panicle each of NSIC Rc160 and NERICA-L-19, which set 1 and 2 seeds, respectively, when grown in an isolated glass house (Additional file 1: Table S1). For restorer lines, BC_2_F_3_ plants of NSIC Rc 160, NSIC Rc 240, Ciherang, BRRI dhan 29, and NERICA-L-19, and plants backcrossed with local Basmati three times followed by backcrossing once with Pusa Basmati showed higher seed setting rates, which values were not significantly different from those of their pollen parents, except for NERICA-L-19 showing variation of lower seed setting rates depending on individual BC_2_F_3_ plants (Additional file 1: Table S1). A seed setting rate of lines backcrossed with Samba Mahsuri was 52.6% for BC_3_F_2_ plants with *rf17rf17*, while 74.0% for BC_2_F_3_ plants with *Rf17Rf17* (Additional file 1: Table S1), suggesting Samba Mahsuri might have a *Rf* gene with weak function of fertility restoration.

To know whether Samba Mahsuri carried another allele of the *Rf17* gene, a 5-kb genomic region including the 3.9-kb promoter and 0.5-kb coding sequences of the *RF17* gene of Samba Mahsuri and IR 64 were amplified by PCR using the following primers: 5′-AAGAGATGACGGTGCAGTTC-3′, 5′-TCGTTCACCACGGTAGATAGACTCAT-3′, 5′-CCCACATCTTCTCCTTGCATAATCC-3′, and 5′-GGGGCTCCCTAGGTGGCTAA − 3′. The nucleotide sequence of Samba Mahsuri was completely identical to that of IR 64. Because IR 64 does not have fertility restoration abilities, the *rf17* allele of IR 64 and Samba Mahsuri was non-functional for fertility restoration. This result indicated that Samba Mahsuri had a new fertility restorer gene different from the *Rf17* gene.

In conclusion, we produced CMS lines of NSIC Rc 160, NSIC Rc 240, Ciherang, BRRI dhan 29, NERICA-L-19, and Pusa Basmati, which did not set any seeds. The fertility was fully recovered by the presence of *Rf17*. The CW-CMS/*Rf17* system will be useful for the production of CMS lines of various Indica Group rice cultivars and for hybrid rice breeding.

## Supplementary information

**Additional file 1: Table S1**. Seed setting rates (%) of backcrossed lines and pollen parents in Tropical Agriculture Research Front, Japan International Research Center for Agricultural Sciences. (PDF 47 kb)

## Data Availability

The datasets supporting the conclusions of this article are included within the article. The nucleotide sequences of the *rf17* gene of Samba Mahsuri and IR 64 have been deposited to DNA Data Bank of Japan under the accession numbers of LC456267 and LC456268.

## Abbreviations

CMS: cytoplasmic male sterility
CW-CMS: Chinese wild rice-type CMS
Rf: Restorer of fertility
WA-CMS: wild-abortive-type CMS

## References

[CR1] Cai J, Liao QP, Dai ZJ, Zhu HT, Zeng RZ, Zhang ZM, Zhang GQ (2013). Allelic differentiation and effects of the *Rf3* and *Rf4* genes on fertility restoration in rice with wild abortive cytoplasmic male sterility. Biol Plant.

[CR2] Fujii Sota, Kazama Tomohiko, Yamada Mari, Toriyama Kinya (2010). Discovery of global genomic re-organization based on comparison of two newly sequenced rice mitochondrial genomes with cytoplasmic male sterility-related genes. BMC Genomics.

[CR3] Fujii S, Toriyama K (2005). Molecular mapping of the fertility restorer gene for ms-CW-type cytoplasmic male sterility of rice. Theor Appl Genet.

[CR4] Fujii S, Toriyama K (2009). Suppressed expression of *RETROGRADE-REGULATED MALE STERILITY* restores pollen fertility in cytoplasmic male sterile rice plants. Proc Natl Acad Sci U S A.

[CR5] Huang JZ, E ZG, Zhang HL Shu QY (2014) Workable male sterility systems for hybrid rice: genetics, biochemistry, molecular biology, and utilization. Rice 2014, 7:13. doi:10.1186/s12284-014-0013-6 10.1186/s12284-014-0013-6 PMC4883997 26055995

[CR6] Jing RC, Li XM, Yi P, Zhu YG (2001). Mapping fertility-restoring genes of rice WA cytoplasmic male sterility using SSLP markers. Bot Bull Acad Sinica.

[CR7] Katsuo K, Mizushima U (1958). Studies on the cytoplasmic difference among rice varieties, Oryza sativa L. 1. On the fertility of hybrids obtained reciprocally between cultivated and wild varieties. Japan J Breed.

[CR8] Kojima Y, Ebana K, Fukuoka S, Nagamine T, Kawase M (2005). Development of an RFLP-based rice diversity research set of germplasm. Breed Sci.

[CR9] Ohnishi T, Yoshino M, Toriyama K, Kinoshita T (2013). Rapid establishment of introgression lines using cytoplasmic male sterility and a restorer gene in Oryza sativa cv. Nipponbare. Mol Breed.

[CR10] Sattari M, Kathiresan A, Gregorio GB, Hernandez JE, Nas TM, Virmani SS (2007). Development and use of a two-gene marker-aided selection system for fertility restorer genes in rice. Euphytica.

[CR11] Toriyama K, Kazama T (2016) development of cytoplasmic male sterile IR24 and IR64 using CW-CMS/Rf17 system. Rice (2016) 9:22 Doi:10.1186/s12284-016-0097-2 10.1186/s12284-016-0097-2 PMC4864779 27167516

[CR12] Virmani Sant S. (1994). Heterosis in Rice. Heterosis and Hybrid Rice Breeding.

