# Supplementary material for: Development of cytoplasmic male sterile lines and restorer lines of various elite Indica Group rice cultivars using CW-CMS/Rf17 system
Source: Rice (N Y). 2019 Sep 18;12:73. doi: 10.1186/s12284-019-0332-8 (PMC6751230; doi:10.1186/s12284-019-0332-8)
Supplement: Supplementary file 1 — Additional file 1: Table S1. Seed setting rates (%) of backcrossed lines and pollen parents in Tropical Agriculture Research Front, Japan International Research Center for Agricultural Sciences. (PDF 47 kb) [file 12284_2019_332_MOESM1_ESM.pdf]

Table S1 Seed setting rates (%) of backcrossed lines and pollen parents in Tropical Agriculture Research Front, Japan International Research Center Agricultural Sciences.

Initial female parent	Recurrent pollen parent	Isolated glasshouse				Open paddy field														
		rf17rf17				*Rf17Rf17*								Pollen parent						
			Individual plants				Individual plants						Average	Individual plants						Average
CWR-IR 64	NSIC Rc160	BC_4F_1	0	0.4	0	BC_2F_3	67.0	70.6	70.4	ND	ND	ND	69.4	89.3	79.5	73.7	77.7	75.9	88.6	79.2
CWR-IR 64	NSIC Rc240	BC_4F_1	0	0	0	BC_2F_3	82.8	74.7	84.1	78.2	84.9	89.2	82.3	89.6	87.1	75.9	84.7	79.5	87.7	83.9
CWR-IR 64	Ciherang	BC_4F_1	ND	ND	ND	BC_2F_3	72.4	88.1	81.7	86.6	92.3	88.9	85.0	90.4	89.0	83.2	80.5	62.0	90.2	82.8
CWR-IR 64	BRRI dhan 29	BC_4F_1	0	0	0	BC_2F_3	83.3	82.3	82.0	75.6	70.1	77.3	78.4	79.2	79.2	86.5	66.0	78.6	82.1	78.5
CWR-IR 64	NERICA-L-19	BC_4F_1	0	0	0.8	BC_2F_3	65.1	65.7	66.7	44.5	37.8	17.6	49.6*	84.1	86.5	79.6	80.3	84.8	81.9	78.2
CWR-IR 64 x Local Basmati BC_3F_3	Pusa Basmati	BC_4F_1	0	0	0	BC_1F_3	78.8	91.7	86.7	85.4	67.8	34.7	74.2	67.6	75.7	63.8	60.4	64.2	48.4	64.5
CWR-IR 64	Samba Mahsuri					BC_2F_3	69.1	71.2	66.4	78.0	85.4	ND	74.0	59.2	69.7	72.4	72.2	81.1	81.1	72.9
						rf17rf17														
CWR-IR 64	Samba Mahsuri	BC_3F_2	ND	ND	ND	BC_3F_2	50.3	39.8	49.4	46.3	62.6	67.1	52.6*							

* indicates that the value is significantly different from the value for its pollen parent ($p < 0.05$).
